# Recombinant Family 1 Carbohydrate-Binding Modules Derived From Fungal Cellulase Enhance Enzymatic Degradation of Lignocellulose as Novel Effective Accessory Protein

**DOI:** 10.3389/fmicb.2022.876466

**Published:** 2022-07-11

**Authors:** Hexue Jia, Xiaoting Feng, Jiamin Huang, Yingjie Guo, Daolei Zhang, Xuezhi Li, Jian Zhao

**Affiliations:** ^1^State Key Laboratory of Microbial Technology, Shandong University, Qingdao, China; ^2^School of Bioengineering, Shandong Polytechnic, Jinan, China

**Keywords:** lignocellulose, enzymatic hydrolysis, family 1 carbohydrate binding module, promoting mechanism, accessory protein

## Abstract

Fungal cellulases usually contain a family 1 carbohydrate-binding module (CBM1), and its role was considered to recognize the substrate specifically. This study testified that the CBM1s derived from cellobiohydrolase I of *Trichoderma reesei, Penicillium oxalicum*, and *Penicillium funiculosum* could be used as an effective accessory protein in cellulase cocktails to enhance the saccharification of lignocellulose, and its enhancement effect was significantly superior to some reported accessory proteins, such as bovine serum albumin (BSA). The promoting effects of the CBM1s were related to not only the CBM1 sources and protein dosages, but also the substrate characteristics and solid consistency during enzymatic hydrolysis. The adsorption capacity of the CBM1s, the adsorption kinetic of TrCBM from *T. reesei* and cellobiohydrolase, endoglucanase, and β-glucosidase from *P. oxalicum*, and the effect of adding TrCBM on enzyme activities of free cellulases in the hydrolysis system were investigated, and the binding conformations and affinities of CBM1s to cellulose and lignin were predicted by molecular docking. It was speculated that the higher affinity of the CBM1s to lignin than cellulases could potentially enable the CBM1s to displace cellulase adsorbed on lignin or to preferentially adsorb onto lignin to avoid ineffective adsorption of cellulase onto lignin, which enhanced cellulase system efficiency during enzymatic hydrolysis of lignocellulose.

## Introduction

A lignocellulosic material is an abundant and low-cost renewable resource on earth, mainly containing cellulose, lignin, and hemicellulose (Himmel et al., 2007). It could be used for producing biofuels and biobased chemicals by enzymatic hydrolysis with cellulase and fermentation, which is of great significance for alleviating environmental and energy problems (Taha et al., 2016). Now, high cellulase cost is one of the major factors influencing the industrial-scale bioconversion of lignocellulose. It is of great significance to develop measures for reducing the high cellulase cost in lignocellulose bioconversion (Gomes et al., 2015).

Many studies reported that some accessory proteins, such as yeast extract, peptone, BSA (bovine serum albumin), non-ionic surfactants, whey protein, and sophorolipid, could coordinate with cellulase or protect cellulase, therefore enhancing the efficiency of cellulase in enzymatic hydrolysis and decreasing the cellulase dosage in the saccharification stage (Dekker, 1983; Ooshima et al., 1986; Yang and Wyman, 2006; Zheng et al., 2008; Kim et al., 2009; Wang et al., 2015; Méndez Arias et al., 2017; Xu et al., 2021). For example, adding yeast extract and peptone to the hydrolysis system could heighten the hydrolysis efficiency of pretreated rice straw (Wang et al., 2015). The additions of expansin and BSA promoted the enzymatic degradation of filter paper (Kim et al., 2009) and cellulose in actual lignocellulosic substrates (Yang and Wyman, 2006; Zheng et al., 2008), respectively. Du et al. reported that BSA addition could dramatically increase the sugar yield of pretreated wheat straw by decreasing the unproductive adsorption of cellulase caused by lignin, thus decreasing enzyme dosage when the same yield of glucose was obtained (Du et al., 2018a). The additions of non-ionic surfactants, such as Tween 20, could improve the conversion of cellulose in pretreated creeping wild ryegrass (Ooshima et al., 1986; Zheng et al., 2008). The additions of whey protein and sophorolipid could reduce the inactivation rate of cellulase under high shear and temperature conditions, thus facilitating the hydrolysis process of pretreated sugarcane bagasse (Xu et al., 2021). Now, the addition of accessory protein is regarded as an important and emerging strategy to increase the hydrolysis efficiency of lignocellulose and reduce enzyme cost. However, more efficient accessory proteins are still needed to be developed for decreasing the addition cost (Kim et al., 2014).

A family 1 carbohydrate-binding module (CBM1) is widely found in fungal cellulases, and it is connected to the catalytic domain of cellulase through linker peptides (Lombard et al., 2014). In general, the primary function of CBM1 is to specifically recognize the insoluble substrate and make the catalytic domain (CD) approach to the substrate, thus increasing the local concentration of cellulase and promoting the degradation of cellulose (Oliveira et al., 2015). The hydrolysis process of lignocellulose material was complex and related to multiple enzyme–substrate interactions. Among them, the adsorption of cellulases, especially cellobiohydrolase (CBH), onto the surface of cellulose was the precondition for efficient hydrolysis of the substrate (Zhai et al., 2018). The hydrophobic interaction mediated by CBM1 was considered the main reason for the binding of cellulases to the substrate (Oliveira et al., 2015). Moreover, it was reported that CBM1 alone could also increase the accessibility of enzyme to cellulose by destroying the compact crystalline structure of cellulose (Wang et al., 2008; Hall et al., 2011; Bernardes et al., 2019). In addition, some research studies also reported the role of CBM1 in the interaction between cellulase and lignin (Linder and Teeri, 1997; Kotiranta et al., 1999; Palonen et al., 2004; Rahikainen et al., 2013a; Gao et al., 2014; Strobel et al., 2016; Lu et al., 2017) and found that CBM1 played a significant function in the adsorption of cellulase onto lignin (Palonen et al., 2004; Lu et al., 2017). Removing CBM1 significantly reduced the affinity of CBHI (cellobiohydrolase I) to lignin (Palonen et al., 2004), and the binding ability of proteins and insoluble lignin was increased when fusing CBM1 to cellulase (Gao et al., 2014; Lu et al., 2017). Based on these research studies, it could be speculated that CBM1 could be used as a novel accessory protein during enzymatic degradation of pretreated lignocellulosic substrates because of the dual effects of destroying the crystal structure of cellulose and acting as a blocker to decrease the unproductive adsorption of cellulase onto lignin. Up to now, however, the published research studies about the function of CBM1 alone in enzymatic hydrolysis were using pure cellulose (such as Avicel) as the model substrate. The possible impact of CBM1, as an isolated accessory protein rather than a part of cellulase, on the enzymatic degradation of pretreated lignocellulosic substrates containing complex components has not been reported.

On the contrary, it has also been reported that the CBM1s of CBHI derived from different strains showed different influences on the enzymatic degradation of pretreated lignocellulose (Du et al., 2018b; Taylor et al., 2018). For example, the CBM of CBHI derived from *Trichoderma reesei* performed better in the degradation efficiency on crystalline cellulose than that from *Penicillium oxalicum* (Du et al., 2018b). Taylor et al. found that, by replacing the CBM1 of CBHI from *Penicillium funiculosum* with the corresponding domain of *T. reesei*, the enzymatic performance of the recombinant CBHI on dilute-acid-pretreated corn stover was slightly reduced (Taylor et al., 2018). Thus, the understanding of different CBM1 functions and their promoting effects on enzymatic hydrolysis contributed to further developing a high-efficiency cellulase mixture to reduce the cost of cellulase used in the bioconversion of lignocellulose.

In this study, the CBM1 genes of CBHI from *T. reesei, P. oxalicum*, and *P. funiculosum* were cloned and subsequently expressed in *Escherichia coli* (named TrCBM, PoCBM, and PfCBM, respectively). The purified CBM1s were added to the hydrolysis system of filter paper (FP) and liquid-hot-water-pretreated corn stover (LPCS) with cellulase, respectively, for studying and comparing their functions in enzymatic degradation of lignocellulose. Adsorption characteristics of the CBM1s on cellulose and lignin and the influences of the additions of the isolated CBM1 proteins on adsorption of cellulase onto substrates were also studied, and the possible mechanism of CBM1 promoting enzymatic degradation of pretreated lignocellulose was further speculated.

## Materials and Methods

### Materials and Strains

Filter paper (FP) was purchased from Aladdin, China. To prepare liquid-hot-water-pretreated corn stover (LPCS), the raw material was cut into 2–3 cm, then treated with water at 150 °C for 1 h, and subsequently washed to neutral with tap water. The chemical components of biomass substrates were analyzed, and the percentage contents of glucan (cellulose), hemicellulose, and lignin based on the weight of dry biomass matter were calculated, respectively, according to the NREL methods (Sluiter et al., 2008).

The cellulase SP used in the hydrolysis process was the commercial cellulase preparation derived from *P. oxalicum* JU-A10, produced by Sino Biotechnology Co., Ltd. (Gansu, China) (Song et al., 2016; Du et al., 2018b).

### Protein Expression and Purification

TrCBM, PoCBM, and PfCBM were derived from CBHI of *T. reesei, P. oxalicum*, and *P. funiculosum* (GenBank: XP_006969224.1, EPS32984.1, and ADX60067.1, respectively). In order to obtain the CBM1 proteins, the genes of CBM1s were fused into pET 32a vector by restricting enzyme digestion and ligation and overexpressed in *E. coli* BL21 (DE3) (Lu et al., 2017). The correctness of sequences was confirmed by Sanger sequencing. The CBM1 proteins were produced according to the pET System Manual and purified using a HisTrapTM-FF column according to the literature (Zou et al., 2013). The empty pET 32a vector that encoded a thioredoxin protein and His6tag, which was named Tag, was also expressed and purified as mentioned above and used as the control for eliminating the interference of the Tag.

Several main cellulase components from *P. oxalicum*, CBH I (Cel7A-2, GenBank: EPS32984.1), EG II (endoglucanase II, Cel5B, GenBank: EPS34262.1), and BG I (β-glucosidase, Cel3A, GenBank: EPS27792.1) were, respectively, expressed in *P. oxalicum* strain A11Δ, a low extracellular background strain as reported in the literature (Hu et al., 2015; Lu et al., 2018; Du et al., 2020). Specifically, the genes of CBH I, EG II, and BG I were ligated into K-hph-p15A vector, and then, the ligated fragments were transferred into A11Δ strain. Proteins were produced by starch induction and purified for further experiments.

### Enzymatic Hydrolysis

The enzymatic hydrolysis was carried out in 125 mL of Erlenmeyer flasks with a reaction system of 40 mL. The pH of reaction solution was adjusted to 4.8 by sodium acetate buffer (0.2 M), and the solid content was 5% dry mass (DM). The reaction conditions of enzymatic hydrolysis were set at 150 rpm and 48°C. The enzyme loading of cellulase SP was 3 mg/g DM, and the protein loading of CBM1s (about 26 kDa), BSA (Sigma, United States, 66 kDa), and Tag (about 22 kDa) was 1.5 mg/g DM, respectively. The CBM1 proteins, BSA, and Tag were premixed with cellulase solution, respectively, and then added to the reaction system of enzymatic hydrolysis according to the experimental designs. The protein concentrations were determined by Bradford protein assay kit, which was purchased from Sangon, Shanghai, China.

After enzymatic hydrolysis of substrates to the specified time, the hydrolysates were taken and centrifuged at 13,000 rpm for 10 min to obtain supernatants of samples and then filtrated by a 0.22-μm filter, and the obtained supernatants were used for analyzing the glucose contents using high-performance liquid chromatography (HPLC, Shimadzu, Kyoto, Japan) with a Bio-Rad HPX-87P column and a Shimadzu refractive index detector. The temperature was set at 78°C, and Milli-Q was selected as eluent, with the flow rate setting to 0.5 mL/min.

The glucan conversion was calculated according to equation (1):


(1) $$Glucanconversion\left(\%\right)=\frac{G\,l\,u\,c\,o\,s\,e\,r\,e\,l\,e\,a\,s\,e\,d\,f\,r\,o\,m\,h\,y\,d\,r\,o\,l\,y\,s\,i\,s\,\left(m\,g\right)}{\begin{array}{c} D\,r\,y\,s\,u\,b\,s\,t\,r\,a\,t\,e\,w\,e\,i\,g\,h\,t\,\left(m\,g\right)\\ \times Glucancontentinsubstrate\left(\%\right) \end{array}}\times 0.9\times 100\%$$

Statistical analysis of standard variance was conducted by the analysis of variance (ANOVA) using the SPSS program (version 22.0).

### Determination of Enzyme Activities in the Supernatant

*p*-Nitrophenyl-D-cellobioside (*p*NPC; Sigma, United States), sodium carboxymethylcellulose (Yuanye Bio-Technology Co., Ltd., Shanghai, China), and *p*-nitrophenyl-β-D-glucopyranoside (*p*NPG; Sigma, United States) were selected to measure the enzymatic activities of cellobiohydrolase (CBH), endoglucanase (EG), and β-glucosidase (BG), respectively. In brief, for determining the enzymatic activities of CBH and BG, 100 μL of diluted enzyme was incubated with 50 μL *p*NPC (gluconolactone was added as an inhibitor) or *p*NPG solution (1 mg/mL) for 30 min at 50°C, and then 150 μL of Na_2_CO_3_ solution (10%, w/w) was added to the reaction system, and the absorbance of mixture at 420 nm was determined. The EG activity was determined by mixing 60 μL of sodium carboxymethylcellulose solution (1%, w/v) and 20 μL of diluted enzyme and then incubating at 50°C for 30 min. DNS method was used to determine the reducing sugar yield in the reaction system (Ghose, 1987). A unit of enzyme activity was defined as the amount of enzyme required to produce 1 μmol of product per minute.

### The Effect of TrCBM Addition on Different Activities of Free Cellulase in Hydrolysis System

Lignin was first extracted from LPCS according to the method reported previously (Lu et al., 2016). In short, the residual sugar content in LPCS was reduced to less than 2% by hydrolyzing LPCS with cellulase and then degrading cellulase using protease to get lignin. Adsorption experiment was conducted in 1 mL of reaction system with acetate buffer (pH 4.8, 0.2 M). The commercial cellulase SP (10 mg protein/g lignin), TrCBM (1 mg protein/g lignin), and lignin (0.03 g) were thoroughly mixed and then incubated for 24 h at 48°C and 150 rpm. After incubation, the mixture system was centrifuged, and the activities of different enzymes in the supernatant were analyzed for studying the enzymatic activity changes caused by TrCBM addition. The reaction system with cellulase SP but not adding TrCBM was used as control.

### Adsorption of Family 1 Carbohydrate-Binding Modules and Cellulases Onto Cellulose and Lignin

The substrate (cellulose or lignin) was mixed with different proteins (CBM1s, CBHI, EGII, or BGI, respectively) in 1 mL of the hydrolysis system with sodium acetate buffer (0.2 M, pH 4.8), and the mixtures were incubated at a constant speed of 150 rpm for 24 h. The adsorption experiments were conducted at 48°C for lignin, but at 4°C for cellulose to avert cellulose degradation. After incubation, supernatants were obtained by centrifugation at 10000 rpm for 10 min. The protein contents in supernatants were determined by the Bradford method (Bradford, 1976), and the quantities of proteins adsorbed on substrates were obtained by calculating the differences in protein amounts in supernatants before and after incubation. For the Langmuir adsorption experiment, cellulose or lignin was quantitative, while the concentrations of TrCBM or cellulases were different. In the experiment comparing the adsorption capacity of different CBM1s, the loading of CBM1s was 10 mg protein/g substrate.

The adsorption data were analyzed and calculated by the Langmuir expression (2):


(2) $$\text{T}=\frac{\text{c}\cdot \text{TMAX}\cdot \text{K}}{\text{c}\cdot \text{K}+1}$$

where *c* represents the protein concentration after incubation (mg/mL), *T* represents the amount of protein adsorbed to the substrate (mg/g), *T*_*MAX*_ represents the maximum adsorption amount of cellulose or lignin, and *K* is the adsorption constant (the higher *K* value, the higher affinity of protein to the substrate). In this formula, *T* and *c* were measured by adsorption experiments and *T*_*MAX*_ and *K* were obtained by fitting.

### Molecular Docking

Cellohexaose and homodimers of guaiacyl (LGG) were selected as ligands (Mertz et al., 2007; Lima et al., 2013). The structures of PoCBM and PfCBM were modeled by SWISS-MODEL server, and TrCBM (PDB: 1cbh) was selected as the template, which was determined by the previous report (Kraulis et al., 1989). The internal water molecules were removed, and hydrogen atoms were increased before molecular docking. AutoDock software was used to dock cellohexaose and LGG with CBM1s, respectively. The CBM1s were set as a rigid body, and the ligands were flexible. In the docking process, the binding conformation and binding energy were calculated by using a Lamarckian genetic algorithm (LGA). PyMOL software was used for structural visualization.

## Results and Discussion

### Expression and Purification of Family 1 Carbohydrate-Binding Modules

**FIGURE 1:**
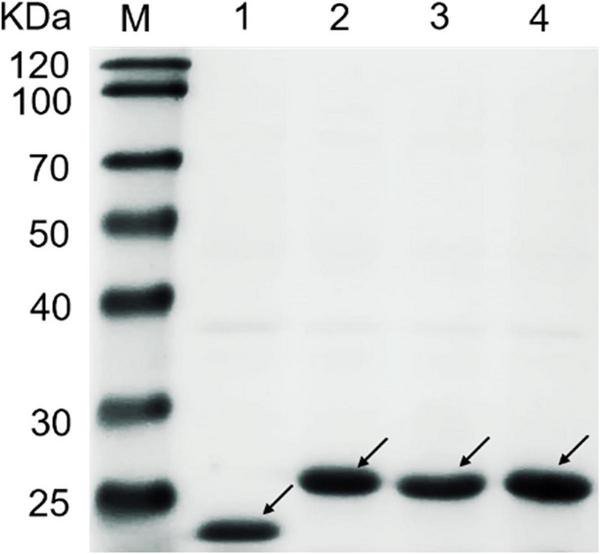
SDS–PAGE analysis of TrCBM, PoCBM, PfCBM, and Tag protein. Lane M, protein marker; Lane 1, Tag; Lane 2, TrCBM; Lane 3, PoCBM; Lane 4, PfCBM.

The CBM1 genes of CBHI derived from *T. reesei, P. oxalicum*, and *P. funiculosum* were, respectively, fused to the pET 32a vector and expressed in *Escherichia coli* BL21 (DE3) (named TrCBM, PoCBM, and PfCBM, respectively). Initially, we tried to express CBM1 alone in *E. coli*, but no protein was detected. It may be due to the low molecular weight of CBM1 (about 4 kDa), leading to rapid degradation in the cell (Cheng and Patel, 2004; Mello and Polikarpov, 2014). Hence, there were a thioredoxin and His6tag encoded on vector, which was named Tag, conducive to the fusion protein expression. In addition, the empty vector with Tag was also expressed in *E. coli* and used for the subsequent saccharification experiment as the control to eliminate the interference of the Tag. All the expressed proteins were purified using a HisTrapTM-FF column, as mentioned in the literature (Zou et al., 2013). The results of SDS–PAGE analysis of TrCBM, PoCBM, PfCBM, and Tag protein are shown in Figure 1. It was shown that the molecular weights of the recombinant CBM1s and expressed Tag were about 26 kDa and about 22 kDa, respectively, close to the predicted molecular weights. The purified CBM1 proteins and Tag protein were used in further saccharification experiments.

### Function of Family 1 Carbohydrate-Binding Modules From Different Sources on Enzymatic Hydrolysis of Filter Paper and Liquid-Hot-Water-Pretreated Corn Stover

#### Promoting Effects of Various Family 1 Carbohydrate-Binding Modules on Enzymatic Hydrolysis

**FIGURE 2:**
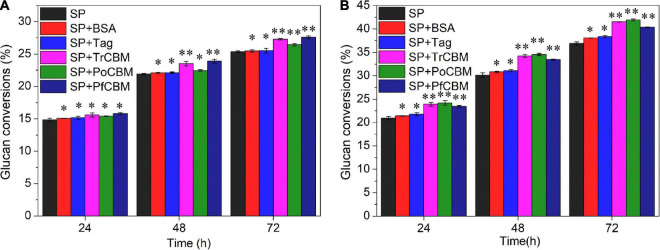
Effect of the addition of BSA, Tag, TrCBM, PoCBM, and PfCBM proteins (1.5 mg/g DM) on glucan conversions of FP **(A)** and LPCS **(B)** at 72 h of enzymatic hydrolysis. ^**^Represents that there was a significant difference (*p* < 0.05), and *means that the difference was not significant (*p* > 0.05).

Filter paper and LPCS were selected as substrates for studying the effects of the isolated CBM1s from different sources on enzymatic degradation of lignocellulose. By analyzing chemical compositions of FP and LPCS, the contents of glucan, hemicellulose, and lignin in LPCS were 58.06, 8.82, and 21.71%, respectively, and FP contained glucan of 92.38%, hemicellulose of 3.63%, and lignin of 0.82%. That is to say, the cellulose was the main component, and there were almost no hemicellulose and lignin in FP. It was found that no glucose was produced when only recombinant CBM1s were used to treat FP or LPCS in the absence of cellulase (results not shown), indicating that the isolated CBM1s alone could not degrade cellulose into fermentable sugar. Further, the recombinant CBM1 proteins, including TrCBM, PoCBM, and PfCBM (protein loading of 1.5 mg/g DM), were added to the degradation systems with commercial cellulase SP (enzyme loading of 3 mg protein/g DM) of FP and LPCS, respectively, for evaluating the effects of CBM1s from different sources on enzymatic hydrolysis of substrates. Meanwhile, BSA and Tag (1.5 mg/g DM) were also, respectively, added to the hydrolysis as control. Glucan conversions at several hydrolysis times were calculated and are shown in Figure 2.

Figure 2A shows that the addition of BSA or Tag had little influence on the conversion rate of glucan in FP, indicating that BSA alone and Tag alone could not degrade cellulose. Yang and Wyman reported the low adsorption capacity of BSA on cellulose (Yang and Wyman, 2006) and thus little influence on quantities of cellulase adsorbed on cellulose in FP. Figure 2A also shows that, compared with several controls (only adding cellulase, adding BSA and adding Tag, respectively), the additions of recombinant CBM1 proteins increased the glucan conversions of FP. For example, the additions of TrCBM, PoCBM, and PfCBM increased glucan conversions at 72 h of enzymatic hydrolysis by 7.58, 4.21, and 8.85%, respectively, when compared with the control that only added cellulase, which showed that the additions of CBM1s could enhance the ability of the cellulase system to degrade FP. Mello and Polikarpov reported that the CBM1 of CBHI from *Trichoderma harzianum* could enhance enzymatic degradation of FP with a commercial cellulase preparation (Mello and Polikarpov, 2014).

Unlike FP, compared with the control that only added cellulase, the additions of BSA and Tag proteins could slightly increase the glucan conversions of LPCS (Figure 2B). For example, glucan conversions of LPCS at 72 h of enzymatic hydrolysis increased by 3.21 and 3.95%, respectively, higher than that of FP, which may be attributed to the differences in the lignin contents between LPCS and FP. It has been reported that the addition of BSA could increase the glucose yield during enzymatic hydrolysis of various pretreated lignocelluloses because BSA can bind to the hydrophobic region of lignin, thus decreasing the non-productive adsorption of cellulase onto lignin (Yang and Wyman, 2006; Wang et al., 2015). Further, it was found that the additions of TrCBM, PoCBM, and PfCBM proteins increased the glucan conversions at 72 h of enzymatic hydrolysis of LPCS by 12.60, 13.69, and 9.38%, respectively, significantly higher than the additions of BSA and Tag. The results indicated that (1) the promoting effects of recombinant CBM1s (Tag-fused CBM1s) on enzymatic degradation were mainly owing to CBM1 of recombinant CBM1s rather than Tag of recombinant CBM1s and (2) the recombinant CBM1s could be used as effective auxiliary proteins to enhance saccharification of pretreated lignocellulose biomass.

Moreover, it can also be discovered in Figure 2 that the promoting effects of isolated CBM1s on enzymatic degradation of lignocellulose were not only related to the types of substrates, but also related to the sources of CBM1s. For example, the addition of the isolated TrCBM protein promoted the enzymatic degradation of FP and LPCS, which may be due to the ability of CBM of CBH1 derived from *T. reesei* to destroy the crystalline structure of cellulose (Hall et al., 2011), increasing the accessibility of cellulase to the substrate. For LPCS, the promoting effect of PoCBM on enzymatic hydrolysis was higher than that of TrCBM and PfCBM, but it was lower for the FP substrate, which may be related to the different adsorption characteristics of the recombinant CBM1 proteins on various substrates. More reasons for this will be investigated in further work.

#### Effect of Protein Dosage and Substrate Concentration on Promoting Effect of TrCBM

**FIGURE 3:**
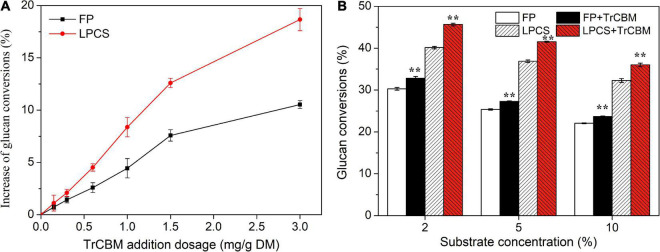
Effect of TrCBM enzyme loading **(A)** and substrate concentrations **(B)** on glucan conversions at 72 h in enzymatic hydrolysis of FP and LPCS. **Represents that there was a significant difference (*p* < 0.05).

As the TrCBM protein had a higher promoting effect on enzymatic degradation of FP and LPCS than PoCBM and PfCBM (Figure 2), we further evaluated the function of TrCBM under different conditions of enzymatic hydrolysis, and the results are shown in Figure 3.

It is shown in Figure 3A that, in the range of TrCBM dosages used in this study, the promoting effect of TrCBM on the degradation of FP and LPCS increased with the increase in TrCBM dosage. However, when the addition exceeded 1.5 mg/g DM, the increasing degree of promoting effect became lower. Relatively, the promoting effect of TrCBM on enzymatic degradation of LPCS was stronger than that of FP, which was consistent with the results in Figure 2. Table 1 shows the enhancement of accessory proteins reported in the literature on enzymatic hydrolysis of pretreated lignocellulosic biomass. As shown in Table 1, by adding yeast extract, peptone, and corn steep liquor to the hydrolysis system of alkaline-pretreated rice straw (addition amount was 50 mg protein/g DM, respectively), the glucose yields at 72 h of enzymatic hydrolysis increased by 13.5, 13.7, and 12.7%, respectively (Wang et al., 2015). Xu et al. also found that the additions of whey protein and sophorolipid (25 mg/g DM) enhanced the glucose yields of alkali-pretreated sugarcane bagasse by 11.9 and 17.8%, respectively (Xu et al., 2021). Compared with the above results, the addition of TrCBM of 3 mg/g DM resulted in 18.66% of the increase in the glucan conversion of LPCS (Figure 3A), indicating that the TrCBM protein was a more efficient accessory protein for enhancing degradation ability of cellulase system from *P. oxalicum* than some accessory proteins reported in the literature. More efficient cellulase system is expected to be obtained by compounding CBM protein, which could be economically produced by constructing the engineering strain producing CBM, or by overexpressing CBM protein in cellulase-producing strains to appropriately increase the ratio of CBM protein in cellulase mixture.

**TABLE 1:** Enhancement efficiency of some reported accessory proteins on enzymatic hydrolysis of lignocellulose.

Additives	Substrate	Results	References
Bovine serum albumin (BSA)	Dilute-sulfuric-acid-pretreated creeping wild ryegrass (8% w/w)	Adding 100 mg/g DM BSA increased 10.67% of cellulose conversions at 72 h.	Zheng et al., 2008
Yeast extract, peptone and corn steep liquor	Alkaline-pretreated rice straw (2% w/v)	13.5, 13.7, and 12.7% improvement in glucose yields after 72-h hydrolysis by adding yeast extract, peptone, and corn steep liquor (1 g/L).	Wang et al., 2015
Bovine serum albumin (BSA)	Alkaline-pretreated sugarcane bagasse (2.5% w/v)	The glucose concentration increased by 14% after 48-h hydrolysis with the addition of BSA (5 mg/g DM)	Méndez Arias et al., 2017
Bovine serum albumin (BSA)	Ammonium-sulfite-pretreated wheat straw (20% w/v)	9.5% improvement in cellulose conversions after 72-h hydrolysis with the addition of BSA (300 mg/g DM)	Du et al., 2018a
Soybean protein	Hydrothermally pretreated sugarcane bagasse (15% w/w)	0.8 g/g DM soybean protein increased 26% of glucose release after 24-h hydrolysis	Brondi et al., 2020
Sophorolipid and whey protein	Alkali-pretreated sugarcane bagasse (20% w/v)	The glucose yield was increased by 17.8 and 11.9% after 120-h hydrolysis with the presence of sophorolipid and whey protein (25 mg/g DM)	Xu et al., 2021
TrCBM	Liquid-hot-water-pretreated corn stover (5% w/v)	18.66% improvement in cellulose conversion after 72-h hydrolysis with 3 mg/g DM TrCBM (Equivalent to 0.15 g/L)	This study
			

Figure 3B shows the effects of substrate concentrations on the promoting ability of TrCBM during the hydrolysis of FP and LPCS, in which the addition of TrCBM was 1.5 mg/g DM. When the substrate concentration improved from 2 to 10%, whether FP or LPCS, the glucan conversion decreased. It may be due to product inhibition and mass transfer problems (Kristensen et al., 2009). However, compared with the control that only added cellulase, the addition of TrCBM could still effectively enhance the enzymatic degradation at high substrate concentration, although its promoting effect decreased. For example, compared with the control, the addition of TrCBM increased the glucan conversions of LPCS by 13.78, 12.60, and 11.60% at the substrate concentrations of 2, 5, and 10%, respectively, and the difference in increase levels between the addition of CBM and control was significant (*p* < 0.01) by statistical analysis. However, there were no significant differences between the increased levels caused by different substrate concentrations (*p* > 0.05).

### Possible Promoting Mechanism of Family 1 Carbohydrate-Binding Module on Enzymatic Hydrolysis

#### Effect of TrCBM Addition on Enzymatic Activities of Free Cellulases in Hydrolysis System

**FIGURE 4:**
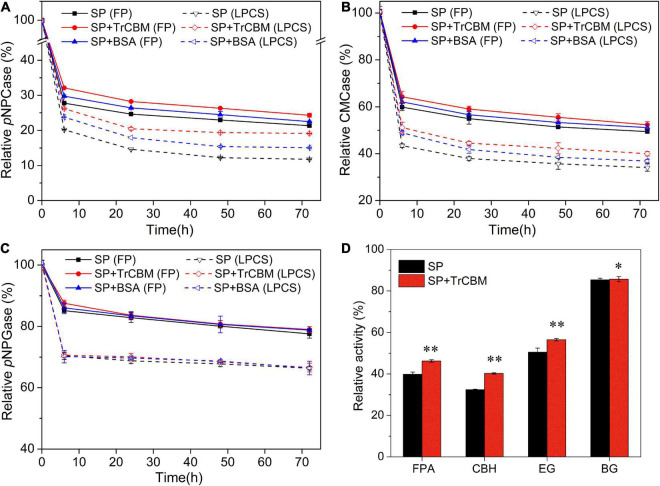
Relative activities of *p*NPCase **(A)**, CMCase **(B)**, and *p*NPGase **(C)** in supernatants from enzymatic hydrolysis systems of FP and LPCS with and without TrCBM addition, and the relative activities of FPA, CBH, EG, and BG after 24 h of lignin adsorption **(D)**. In this, the relative activity of cellulase at 0 h of enzymatic hydrolysis was defined as 100%. ^**^Represents that there was a significant difference (*p* < 0.05), and *means that the difference was not significant (*p* > 0.05).

Using the TrCBM protein as a representative, we investigated the effect of adding isolated TrCBM to cellulases on the cellulase activities in the hydrolysis system by assaying several activities of cellulase in supernatants from enzymatic hydrolysis system of FP and LPCS with and without TrCBM. The hydrolysis system with BSA was used as control. Figure 4 shows the changes in several activities of cellulases in the supernatants at different times of enzymatic hydrolysis.

It is found from Figure 4 that, in the initial phase of enzymatic hydrolysis of FP and LPCS, several activities of cellulases in the supernatants decreased rapidly, regardless of whether TrCBM or BSA was added, which should be attributed to the rapid physical adsorption of proteins onto substrates. Cai et al. (2020) also found that the total cellulases in hydrolysate were decreased quickly after 2 h of enzymatic digestibility of dilute-acid-pretreated eucalyptus. With the increase in hydrolysis time, the enzyme activities in the supernatant further decreased. For example, in the hydrolysis system of FP with cellulase alone, the activities of CBH, EG, and BG in supernatant at 72 h of enzymatic hydrolysis reduced to 21.4, 49.5, and 77.6% of the initial amount, respectively. This phenomenon may be due to partial enzyme inactivation. The activity of CBH in supernatant reduced the most, which might be owing to the high proportion of CBH in the cellulase system from *P. oxalicum* (Song et al., 2016). The lowest reduction in the activity of BG should be owing to the low affinity of BG to the substrate as the absence of CBM (carbohydrate-binding module) in BG.

As shown in Figure 4, in the hydrolysis systems of both FP and LPCS, the addition of BSA increased the relative enzymatic activities of CBH and EG in supernatants. For example, at 72 h of enzymatic hydrolysis, compared with the reaction system with only cellulase, the addition of BSA increased the relative activities of CBH and EG by 5.4 and 3.3%, respectively, for the hydrolysis system of FP and by 28.1 and 8.1% for the hydrolysis system of LPCS. The greater improvement in the enzymatic activities of cellulases in the reaction system of LPCS by adding BSA should be due to the higher content of lignin in LPCS compared with FP. BSA could competitively bind to lignin through hydrophobic force, thus decreasing adsorption of cellulases onto lignin (Lu et al., 2017). In the substrate with high lignin content, this effect of BSA will be more obvious.

Compared with BSA, the addition of TrCBM could more significantly increase the enzymatic activities of free cellulases in supernatant. For example, at 72 h of enzymatic hydrolysis, compared with adding BSA, the addition of TrCBM resulted in the relative activities of CBH and EG in the reaction system of FP increasing by 8.01 and 2.42%, respectively, and by 26.83 and 8.58%, respectively, in the reaction system of LPCS. The more significant enhancement of TrCBM on the enzymatic activities of free CBH and EG in the hydrolysis system should be a reason why the addition of TrCBM had a higher promoting effect on enzymatic degradation of lignocellulose than the addition of BSA (Figure 2). In addition, Figure 4C shows that the additions of TrCBM and BSA had little influence on the enzyme activity of BG in the hydrolysis systems of both FP and LPCS, which was consistent with the results reported by Liuzzi et al. (2019). It was reported by Liuzzi et al. that the addition of BSA increased the total activity of supernatant by about 50% in enzymatic degradation of steam-pretreated corn stover, but had a weak effect on the enzyme activity of β-glucosidase (Liuzzi et al., 2019). Part of the reason may be the lower adsorption capacity of BG on the substrate compared with other cellulases, such as CBH and EG. Some research studies also reported that the adsorption of BG on the substrate was less than that of CBH and EG due to the absence of CBM domain in BG (Várnai et al., 2011; Pribowo et al., 2012).

The presence of lignin affects enzymatic hydrolysis of lignocellulose because lignin not only hinders cellulase access to cellulose as physical barrier, but also adsorbs cellulase through non-productive binding with enzyme, resulting in the enzyme activity loss (Linder and Teeri, 1997; Palonen et al., 2004; Gao et al., 2014; Lu et al., 2017). Therefore, lignin was further extracted from LPCS for studying the effect of TrCBM addition on adsorption of cellulases onto lignin. Figure 4D shows that the addition of TrCBM improved the activities of FPase, CBH, and EG in supernatant after lignin adsorption, especially for CBH activity and EG activity, but little affected the BG activity, consistent with the changes in the enzyme activities in Figures 4A–C. All these results demonstrated that adding TrCBM to the hydrolysis system could reduce the non-productive adsorption of cellulases onto lignin and increase the proportion of free cellulases in the reaction system, thus enhancing enzymatic degradation of lignocellulosic substrate.

Further, the adsorption properties of several main cellulases in the secretome of *P. oxalicum* such as CBHI, EGII, and BGI (Liu et al., 2013; Du et al., 2020) and TrCBM protein onto cellulose and lignin were studied and compared. Langmuir adsorption isotherm was extensively used to compare the kinetic properties of the enzyme–substrate system (Qi et al., 2011; Zheng et al., 2013).

**FIGURE 5:**
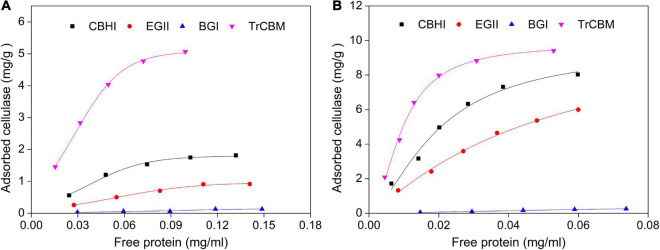
Langmuir isotherm adsorption models of CBHI, EGII, and BGI from *P. oxalicum* and TrCBM on FP **(A)** and lignin extracted from LPCS **(B)**.

Filter paper and lignin extracted from LPCS were used as substrates, respectively, and the adsorption isotherms and parameters of cellulases and isolated TrCBM protein on FP and lignin are shown in Figure 5 and Table 2, respectively. It was shown that the adsorption constants (K values) of CBHI on FP and lignin were 4.85 and 15.00 mL/mg, respectively, higher than that of EGII (2.80 and 11.84 mL/mg, respectively) and BGI (0.96 and 2.39 mL/mg, respectively), which indicated that the affinities of CBHI to cellulose and lignin were higher than those of EGII and BGI. It was consistent with the result that more CBH was adsorbed onto substrates during enzymatic hydrolysis (Figure 4). Guo et al. (2014) also found that the order of adsorption capacity of different cellulase components onto lignin was, in turn, CBH > EG > BG. Moreover, the adsorption constants of the TrCBM protein on FP and lignin were 8.78 and 20.74 mL/mg, respectively, significantly higher than that of CBHI, EGII, and BGI. According to the principle of the Vroman effect, during the adsorption of lignocellulosic degrading enzyme mixture onto substrates, the enzymes with the higher affinity to the substrate could displace the enzymes with the lower affinity (Sammond et al., 2014; Yarbrough et al., 2015). It could be inferred that the TrCBM protein with the higher affinity to lignin could partly displace cellulase components adsorbed on lignin, leading to the increase in free cellulases concentration during enzymatic hydrolysis of lignocellulose.

**TABLE 2:** Parameters of Langmuir isotherm adsorption models of CBHI, EGII, BGI, and TrCBM on FP and lignin extracted from LPCS.

Substrates	Proteins	T_*MAX*_ (mg/g)	K (mL/mg)	*R* ^2^
FP	CBHI	5.49	4.85	0.98
	EGII	3.65	2.80	0.98
	BGI	1.01	0.96	0.96
	TrCBM	12.38	8.78	0.99
Lignin	CBHI	19.61	15.00	0.98
	EGII	14.56	11.84	0.99
	BGI	1.65	2.39	0.98
	TrCBM	25.38	20.74	0.99

#### The Adsorption Capacities of Different Family 1 Carbohydrate-Binding Modules Onto Cellulose and Lignin

**FIGURE 6:**
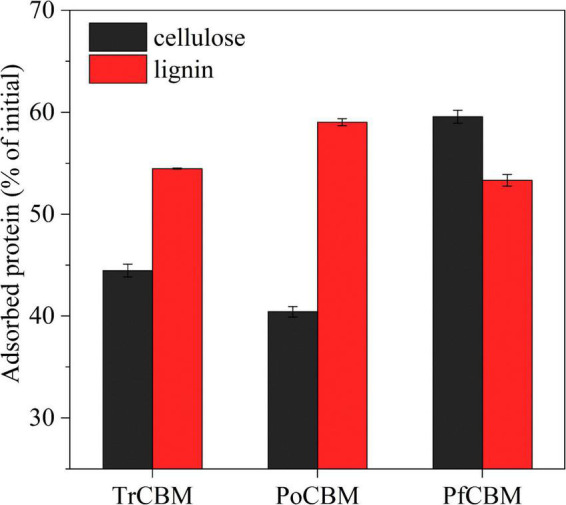
Adsorption capacities of TrCBM, PoCBM, and PfCBM onto FP and lignin extracted from LPCS, in which adsorption time was 24 h.

Pribowo et al. (2012) reported that different enzymes in the cellulase mixture, for example, Cel7A, Cel7B, Cel5A, Xyn10A, and β-glucosidase from *T. reesei*, had different adsorption properties during the hydrolysis of pretreated corn stover, which were largely related to the adsorption properties of the CBMs of the cellulases. Arola and Linder (2016) compared the adsorption affinity of isolated CBM domains, respectively, derived from Cel7A and Cel6A from *T. reesei* to cellulose nanofibril and bacterial microcrystalline cellulose and found that the CBM of Cel7A had the higher affinity to the two substrates than the CBM of Cel6A. In their studies, however, CBM was regarded as a part of enzyme protein structure to discuss the function of CBM domain in adsorption of cellulases with CBM onto substrates. And, the effect of isolated CBM, as an accessory protein, on enzymatic hydrolysis was not involved. In this study, to explain the possible reasons for the additions of isolated CBM1 proteins to promote enzymatic hydrolysis and for the differences in promoting effects of different sources CBM1s from one point of view, we also evaluated the adsorption capacities of the isolated CBM1s from different sources on cellulose and lignin, and the results are shown in Figure 6.

It was found that the order of the adsorption capacity onto cellulose was PfCBM > TrCBM > PoCBM, which was consistent with the order of promoting effect of different CBM1s on enzymatic hydrolysis of FP (Figure 2A). Bernardes et al. reported that CBM1 could be bound to cellulose defects (turns, bends, cracks between fibers, etc.), preventing the ineffective combination of cellulases on cellulose defects, thereby improving the free cellulases concentration in hydrolysate (Bernardes et al., 2019). Similarly, due to the higher affinity to cellulose, more PfCBM may be able to preferentially adsorb onto cellulose defects during enzymatic hydrolysis of FP compared with TrCBM and PoCBM, potentially decreasing the ineffective adsorption of cellulases onto the cellulose defects, thus leading to the greater enhancement on enzymatic hydrolysis of FP (Figure 2A). Moreover, Hall et al. reported that CBM1 from *T. reesei* could non-hydrolytically destroy the dense crystal structure of cellulose (Hall et al., 2011), which heightened the accessibility of enzyme to cellulose. It may be the reason why TrCBM had a higher promoting effect on enzymatic hydrolysis of FP than PoCBM.

It is also found from Figure 6 that the order of the adsorption capacity of different CBM1s onto lignin was PoCBM > TrCBM > PfCBM, which was also consistent with the order of promoting effect of different CBM1s on enzymatic hydrolysis of LPCS (Figure 2B). During enzymatic hydrolysis of the substrate containing lignin, cellulase was adsorbed onto lignin surface through non-specific binding (Rahikainen et al., 2013b), which was the main reason for low degradation efficiency of cellulase (Gao et al., 2014; Liu et al., 2016). Compared with TrCBM and PfCBM, PoCBM with the higher affinity to lignin could be more competitively adsorbed onto lignin, avoiding the non-productive adsorption of cellulase onto lignin and resulting in more promoting effect on enzymatic degradation of LPCS.

#### Prediction of Binding Conformation and Affinity of Different Family 1 Carbohydrate-Binding Modules

**FIGURE 7:**
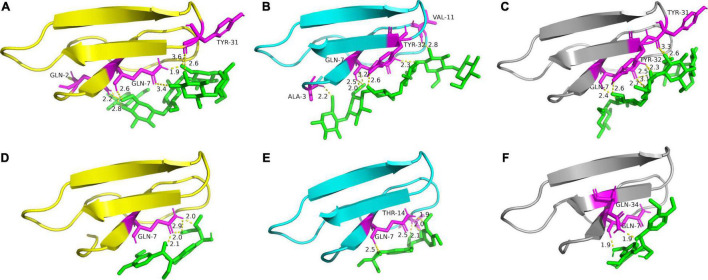
Binding conformations of cellohexaose to TrCBM **(A)**, PoCBM **(B)**, and PfCBM **(C)**, and the conformations of LGG to TrCBM **(D)**, PoCBM **(E)**, and PfCBM **(F)**. The interacted amino acids are shown in magenta.

AutoDock was applied extensively for the prediction of the noncovalent binding between macromolecule (receptor) and small molecule (ligand) and could reflect the binding conformation and affinity (Mertz et al., 2007; Lima et al., 2013; Lu et al., 2018). Cellooligosaccharides and homodimers of guaiacyl (LGG) have been, respectively, docked into the domains of glycoside hydrolase to predict the binding sites of protein with cellulose and lignin by AutoDock (Mertz et al., 2007; Lima et al., 2013; Lu et al., 2017). In this study, the binding conformations and affinities of different CBM1s to cellulose and lignin were predicted by using cellohexaose and LGG to dock with TrCBM, PoCBM, and PfCBM, respectively. Table 3 shows the interaction energies and interacted amino acids at the optimal binding sites of CBM1s with cellohexaose and LGG, and Figure 7 shows the binding conformations of cellohexaose and LGG to different CBM1s.

**TABLE 3:** The interacted energies and amino acids at the optimal binding sites of cellohexaose and LGG to CBM1s.

Ligand	Cellohexaose			LGG		
Macromolecule	TrCBM	PoCBM	PfCBM	TrCBM	PoCBM	PfCBM
Binding energy	−3.74	−3.89	−4.36	−1.91	−2.93	−1.00
Van der Waals–hydrogen bond–desolvation energy	−8.40	−8.53	−9.01	−6.45	−7.62	−5.67
Electrostatic energy	−0.11	−0.14	−0.12	−0.23	−0.08	−0.10
Interacted amino acids	Gln2 Gln7 Tyr31	Ala3 Gln7 Val11 Tyr32	Gln7 Tyr31 Tyr32	Gln7	Gln7 Thr14	Gln7 Gln34

As shown in Table 3, both van der Waals–hydrogen bond–desolvation energy and electrostatic energy played a considerable function in the combinations of all the CBM1s to cellulose and lignin, and the Gln7 (Glutamine) was a critical amino acid that binds CBM1 to both cellulose and lignin, while Tyr31 and Tyr32 (Tyrosine) were important for the binding of CBM1s and cellulose. Beckham et al. also reported that aromatic amino acids, namely, Tyr31 and Tyr32, were critical in the recognition and binding of CBM1 to the substrate (Beckham et al., 2010).

It is found from Table 3 that the binding energies of cellohexaose to TrCBM, PoCBM, and PfCBM were −3.74, −3.89, and −4.36 kcal/mol, respectively. The lower the binding energy between ligand and receptor, the stabler the formed complex (Lu et al., 2017). The lowest binding energy of cellohexaose with PfCBM implied that the PfCBM was easier to bind with cellulose to form a stable complex and thus could potentially release more cellulases adsorbed on the cellulose. It was consistent with the results that the addition of PfCBM had a higher promoting effect on the enzymatic degradation of FP than TrCBM and PoCBM (Figure 2A). However, the binding energy of PoCBM to cellohexaose was lower than that of TrCBM, which was inconsistent with the results that TrCBM had greater adsorption capacity on cellulose (Figure 6) and higher promoting effect on enzymatic hydrolysis of FP (Figure 2A). Hall et al. found that the CBM1 from *T. reesei* could disrupt the crystalline structure of cellulose, therefore improving the accessibility of cellulase to the substrate (Hall et al., 2011). It is also shown in Figure 7 that, when cellohexaose interacted with TrCBM, its binding had obvious rotation, but the binding between cellohexaose and PoCBM had almost no rotation. The rotation of polysaccharide could cause the destruction of hydrogen bonds between the cellulose chains (Wang et al., 2008; Lu et al., 2017), which may partly explain the reason why TrCBM has a higher promoting effect on enzymatic hydrolysis of FP than PoCBM (Figure 2A). In addition, Table 3 shows that the binding energies of LGG to TrCBM, PoCBM, and PfCBM were −1.91, −2.93, and −1.0 kcal/mol, respectively. The lowest binding energy of LGG with PoCBM implied that the PoCBM was easier to bind with lignin to form a stable complex, which may be able to prevent the adsorption of cellulases onto lignin by competitively adsorbing to lignin or release more cellulases adsorbed on the lignin to the reaction system by displacement. It was consistent with the order of adsorption capacities of different CBM1s on lignin (Figure 6) and the order of promoting effect of CBM1s on enzymatic degradation of LPCS (Figure 2B).

## Conclusion

The CBM1s derived from CBHI of *T. reesei, P. oxalicum*, and *P. funiculosum* could be used as a novel accessory protein to enhance the degradation ability of cellulase system from *P. oxalicum*, and their promoting effects on enzymatic hydrolysis of lignocellulose were significantly superior to some accessory proteins reported in the literature. The higher affinity of the CBM1s to lignin than cellulase components such as CBH and EG could potentially enable the CBM1s to displace part of cellulases adsorbed onto lignin or to preferentially adsorb to the lignin for preventing the non-productive adsorption of cellulases onto lignin. The promoting effects of CBM1s were not only related to the CBM1s sources, but also related to substrates characteristics. These findings were helpful in modifying the current cellulase system to improve its catalytic efficiency and reduce the enzyme cost in the bioconversion of lignocellulose.

## Data Availability Statement

The original contributions presented in this study are included in the article/Supplementary Material, further inquiries can be directed to the corresponding author/s.

## Author Contributions

HJ, XL, and JZ conceived the ideas and designed the experiments. HJ, XF, and JH conducted the experiments. HJ, YG, and DZ analyzed the data. HJ wrote the manuscript. JZ revised the manuscript. All authors contributed to the article and approved the submitted version.

## Conflict of Interest

The authors declare that the research was conducted in the absence of any commercial or financial relationships that could be construed as a potential conflict of interest.

## Publisher’s Note

All claims expressed in this article are solely those of the authors and do not necessarily represent those of their affiliated organizations, or those of the publisher, the editors and the reviewers. Any product that may be evaluated in this article, or claim that may be made by its manufacturer, is not guaranteed or endorsed by the publisher.

## Abbreviations

CBM1: family 1 carbohydrate-binding module
BSA: bovine serum albumin
FP: filter paper
LPCS: liquid-hot-water-pretreated corn stover
CBH: cellobiohydrolase
EG: endoglucanase
BG: β-glucosidase
HPLC: high-performance liquid chromatography
CMC: sodium carboxymethylcellulose
*p*NPG: *p*-nitrophenyl - β-D -glucopyranoside
FPA: filter paper activity
*p*NPC: *p*-nitrophenyl -D-cellobioside
LGG: homodimers of guaiacyl
LGA: Lamarckian genetic algorithm
DM: dry mass.
