# Supplementary figures and images for: Recombinant Family 1 Carbohydrate-Binding Modules Derived From Fungal Cellulase Enhance Enzymatic Degradation of Lignocellulose as Novel Effective Accessory Protein
Source: Front Microbiol. 2022 Jul 11;13:876466. doi: 10.3389/fmicb.2022.876466 (PMC9309510; doi:10.3389/fmicb.2022.876466)

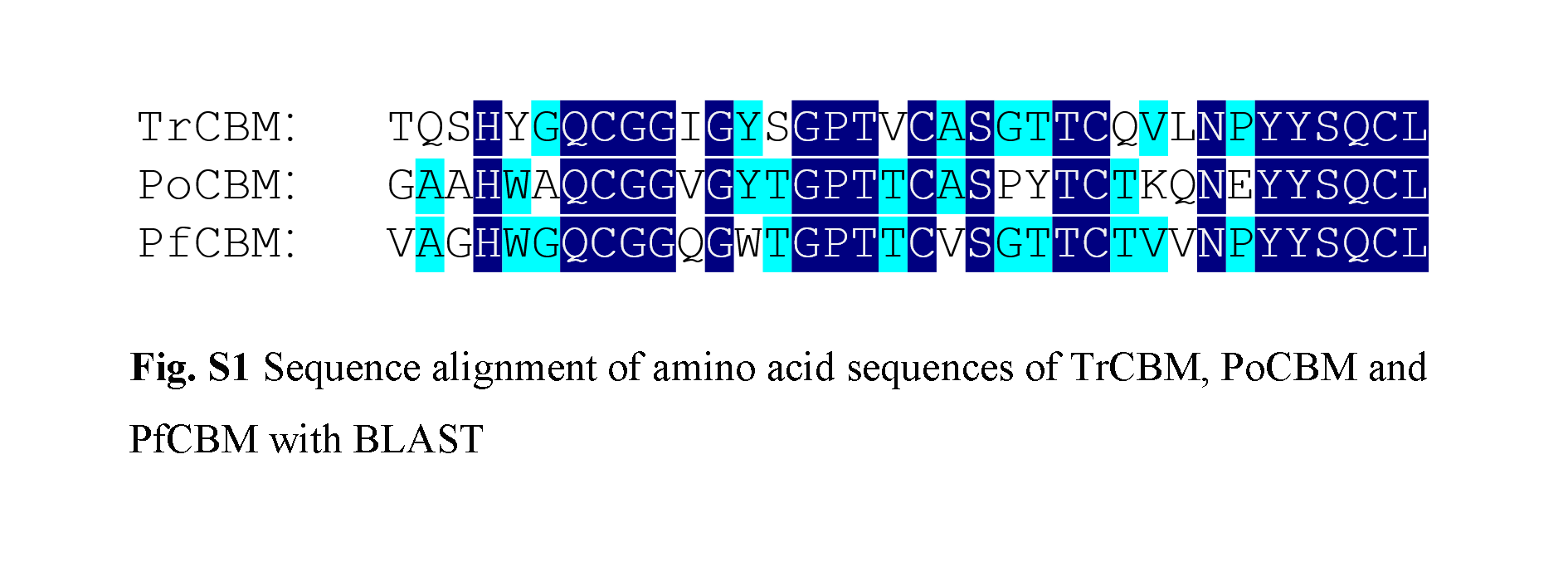

Supplement: Supplementary file 1 [file Image_1.TIF]
